# Prevalence of and factors associated with self-reported high blood pressure in Brazilian adults

**DOI:** 10.1590/S1518-8787.2017051000006

**Published:** 2017-06-01

**Authors:** Deborah Carvalho Malta, Regina Tomie Ivata Bernal, Silvânia Suely Caribé de Araújo Andrade, Marta Maria Alves da Silva, Gustavo Velasquez-Melendez

**Affiliations:** I Departamento de Enfermagem Materno Infantil e Saúde Pública. Escola de Enfermagem. Universidade Federal de Minas Gerais. Belo Horizonte, MG, Brasil; II Núcleo de Pesquisas Epidemiológicas em Nutrição e Saúde. Faculdade de Saúde Pública. Universidade de São Paulo. São Paulo, SP, Brasil; III Departamento de Vigilância de Doenças e Agravos Não Transmissíveis e Promoção da Saúde. Secretaria de Vigilância em Saúde. Ministério da Saúde. Brasília, DF, Brasil; IV Hospital das Clínicas. Universidade Federal de Goiás. Goiânia, GO, Brasil

**Keywords:** Adult, Hypertension, epidemiology, Diagnostic Self Evaluation, Risk Factors, Socioeconomic Factors, Health Surveys, Adulto, Hipertensão, epidemiologia, Autoavaliação Diagnóstica, Fatores de Risco, Fatores Socioeconômicos, Inquéritos Epidemiológicos

## Abstract

**OBJECTIVE:**

To analyze factors associated with self-reported high blood pressure among adults in Brazilian state capitals.

**METHODS:**

The study uses data from Sistema de Vigilância de Fatores de Risco e Proteção para Doenças Crônicas por Inquérito Telefônico (Vigitel – Surveillance System of Risk and Protection Factors of Noncommunicable Diseases by Telephone Survey) collected in 2013. Prevalence rates and their respective 95% confidence intervals by gender were estimated according to sociodemographic variables, lifestyle, reported noncommunicable diseases and self-rated health status. Multivariate logistic regression modeling was used to identify variables associated with self-reported high blood pressure with α < 0.05.

**RESULTS:**

Prevalence of self-reported high blood pressure among adults living in Brazilian state capitals and the Federal District was 24.1%. The following variables were associated with self-reported high blood pressure: age group, taking 18-24 as reference (all age groups presented increased risk – from 25-34 years [OR = 2.6; 95%CI 2.0–3.4] up to 65 years or more [OR = 28.1; 95%CI 21.7–36.4]); low education level (9 to 11 years of study [OR = 0.8; 95%CI 0.7–0.9] and 12 years or more [OR = 0.6; 95%CI 0.6–0.7]); Black race or skin color (OR = 1.3; 95%CI 1.1–1.5); being a former smoker (OR = 1.2; 95%CI 1.1–1.3); obesity (OR = 2.7; 95%CI 2.4–3.0); diabetes (OR = 2.9; 95%CI 2.5–3.5%), and high cholesterol (OR = 1.9; 95%CI 1.8–2.2).

**CONCLUSIONS:**

Approximately one quarter of the adult population living in Brazilian state capitals reported having high blood pressure. Information from Vigitel is useful to monitor high blood pressure and identity its associated factors, supporting public policies for health promotion, surveillance and care.

## RESUMO

**OBJETIVO:**

Analisar os fatores associados à hipertensão arterial autorreferida entre adultos nas capitais brasileiras.

**MÉTODOS:**

Estudo com os dados do Sistema de Vigilância de Fatores de Risco e Proteção para Doenças Crônicas por Inquérito Telefônico (Vigitel) coletados no ano de 2013. Foram estimadas as prevalências e seus respectivos intervalos de confiança 95% por sexo segundo variáveis sociodemográficas, estilos de vida, doenças crônicas relatadas e avaliação do estado de saúde. Modelagem de regressão logística multivariada foi utilizada para identificar as variáveis associadas à hipertensão arterial autorreferida com α < 0,05.

**RESULTADOS:**

A prevalência de hipertensão arterial autorreferida entre os adultos residentes nas capitais brasileiras e Distrito Federal foi de 24,1%. Foram identificadas as seguintes associações com hipertensão arterial autorreferida: faixa etária, tomando 18 a 24 anos como referência, todas as faixas etárias apresentaram maior chance – de 25 a 34 anos (RC = 2,6; IC95% 2,0–3,4) até 65 anos ou mais (RC = 28,1; IC95% 21,7–36,4); baixa escolaridade (9 a 11 anos de estudo – RC = 0,8; IC95% 0,7–0,9; e 12 anos ou mais – RC = 0,6; IC95% 0,6–0,7); raça/cor da pele preta (RC = 1,3; IC95% 1,1–1,5); ser ex-fumante (RC = 1,2; IC95% 1,1–1,3); obesidade (RC = 2,7; IC95% 2,4–3,0); diabetes (RC = 2,9; IC95% 2,5–3,5); e colesterol elevado (RC = 1,9; IC95% 1,8–2,2).

**CONCLUSÕES:**

Cerca de um quarto da população adulta residente nas capitais brasileiras refere ter hipertensão arterial. As informações do Vigitel são úteis para o monitoramento da hipertensão arterial e identificação de seus fatores associados, subsidiando políticas públicas de promoção, vigilância e atenção à saúde.

## INTRODUCTION

Noncommunicable diseases (NCDs) are the main cause of morbidity and mortality, accounting for 63% of causes of death worldwide. Among them, cardiovascular diseases (high blood pressure, infarction, stroke) stand out for their magnitude, accounting for approximately one third of global deaths^a,b^. High blood pressure is the most prevalent circulatory disorder and is often associated with metabolic alterations, which lead to a higher risk for the development of fatal and non-fatal cardiovascular diseases, kidney failure and others^1^.

The World Health Organization (WHO) estimates that about 600 million people are affected by systemic arterial hypertension (SAH) and 7.1 million deaths result from this disease per year. Studies indicate a worldwide growth of 60% of cases by 2025^a^. High blood pressure leads to an increase in health system costs and has affected the global economy^2,3^.

The risk factors associated with arterial hypertension AH described in the literature include inadequate eating habits, excessive salt intake, alcohol abuse, physical inactivity, overweight, smoking, and disorders of the glucose and lipid metabolisms^1,4,5^. AH is a multifactorial clinical condition characterized by high and sustained blood pressure (BP) levels above age-appropriate numbers^b^. There are numerous difficulties in measuring BP at the population level, and therefore most studies use self-reported information as a proxy for actual BP^6^. In the United States, a study comparing the self-reported AH results of the telephone survey Behavioral Risk Factor Surveillance System (BRFSS) with measurement figures from the National Health and Nutrition Examination Survey III show good sensitivity and specificity of self-reported figures^c^.

In Brazil there are few population-based surveys related to AH prevalence. Most studies have limited comparability due to their local or regional scope and differences in questions and methods. Household surveys in Brazilian municipalities estimate prevalence rates ranging from 15% to 40% in the Brazilian urban adult population^7,8^, depending on the methodology and scope of the study. Data from the 2013 National Health Survey (PNS), using self-reported information, estimate a 21.4% prevalence of high blood pressure for the whole country^d^.

Population surveys with probability sampling are costly and carried out over large time intervals. The *Sistema de Vigilância de Fatores de Risco e Proteção para Doenças Crônicas por Inquérito Telefônico* (Vigitel – Surveillance System of Risk and Protection Factors of Noncommunicable Disease by Telephone Survey) has been carried out in the 26 Brazilian state capitals and the Federal District since 2006 and collects information on adult lifestyle and self-reported morbidity, among other topics. It enables annual monitoring of indicators related to NCDs and associated factors to support public policies for health prevention and promotion^e^.

This study aimed to analyze factors associated with high blood pressure in the adult population in the Brazilian state capitals.

## METHODS

The study used Vigitel data collected via telephone survey in 2013 in the adult population (aged ≥ 18 years) living in the 26 state capitals and the Federal District. This system uses probability samples of the adult population based on the cities’ residential landline registries and post-stratification weights calculated by raking^e^ .

These weights aim to adjust the sociodemographic distributions of the sample to the distribution estimated for the total population of 2013. In calculating the post-stratification weights, the weight of the sample was taken as the inverse of the number of telephone lines and individuals in the household. The post-stratification weight is used to generate all the estimates provided by the system for each one of the cities and for those cities as a whole^e^. In 2013, Vigitel interviewed 52,929 adults, about 1,960 in each state capital.

The Vigitel questionnaire includes approximately 94 questions, divided into modules: demographic and socioeconomic characteristics of individuals, eating and physical activity patterns, reported weight and height, reported morbidity, among others^e^.

The outcome analyzed in our study was prevalence of self-reported high blood pressure (HBP), based on a positive response to the question: “Has a doctor ever informed you that you have high blood pressure?”

The explanatory variables were: a) sociodemographic characteristics: gender, age group (18–24, 25–34, 35–44, 45–54, 55–64, 65 or over), schooling (0 to 8 years, 9 to 11 years, 12 years or more), race/skin color (white, black, brown), having health insurance (yes, no); b) lifestyle elements as risk factors: smoking (nonsmoker, former smoker, smoker), body mass index classification (normal, overweight, obese), consumption of fatty red meat (yes, no), alcohol abuse (yes, no), high salt intake (yes, no), insufficient physical activity in the domains “leisure,” “work,” “transport,” and “domestic” (yes, no); free time physical activity – at least 150 minutes of moderate-intensity physical activity per week or 75 minutes of moderate physical activity per week – (yes, no); c) protection factor: recommended consumption of fruit and vegetables – five or more portions per day (yes, no); d) self-reported noncommunicable disease: diabetes (yes, no) and high cholesterol (yes, no); e) self-rated health status (good, fair, poor/very poor).

Prevalence rates of high blood pressure and their 95% confidence intervals (95%CI) were estimated according to the aforementioned explanatory variables. To test the association between the explanatory variables and prevalence of self-reported high blood pressure, a bivariate analysis was first carried out using a 5% significance level test of independence. The variables that showed statistically significant association were selected for the multivariate analysis by logistic regression, using the backward method as selection criterion of variables and 5% significance level for excluding the variable from the model. Data processing and statistical analysis were performed using Stata software version 12.1 (StataCorp., CollegeStation, USA). Odds ratio was used to measure the associations, and sociodemographic variables (race/skin color, health insurance, age, schooling, gender) and metabolic risk factors (diabetes, cholesterol, obesity), eating habits (consumption of fatty red meat, fruit, vegetables, salt intake), alcohol abuse, physical activity and smoking were initially used in model 1. The variable self-rated health status showed collinearity with other variables and therefore was removed from model 1. In model 2, the non-significant variables tested in model 1 were excluded.

The study was approved by the National Human Research Ethics Committee (Opinions 13081/2008 and 355,590/2013).

## RESULTS

Prevalence of self-reported AH among adults (aged ≥ 18 years) living in the Brazilian state capitals and the Federal District was 24.1% (95%CI 23.4–24.8), being higher in women (26.3%; 95%CI 25.4–27.3) and progressing with age, reaching a prevalence rate of 60.4% (95%CI 58.3–62.4) among adults aged 65 and over. Self-reported AH is higher in individuals with lower schooling levels (38.0%; 95%CI 36.5–39.5) and no health insurance (25.3%; 95%CI 24.2–26.3). Participants describing themselves as brown-skinned showed the lowest prevalence of self-reported AH (21.4%; 95%CI 20.3–22.6) (Table 1).

**Table 1:** Prevalence of self-reported high blood pressure and 95%CI in adults (aged ≥ 18 years) according to sociodemographic factors for the Brazilian state capitals and Federal District. Vigitel, 2013.

Variable	%^a^	95%CI	p^b^
Total	24.09	23.39−24.79	
Gender			< 0.001
Male	21.5	20.4−22.5	
Female	26.3	25.4−27.3	
Age group (years)			< 0.001
18−24	3.0	2.4−3.6	
25−34	8.1	7.1−9.1	
35−44	18.3	16.8−19.8	
45−54	34.1	32.2−36.0	
55−64	50.3	48.1−52.5	
65 or over	60.4	58.3−62.4	
Schooling (years)			< 0.001
0−8	38.0	36.5−39.5	
9−11	17.1	16.2−17.9	
12 or over	14.6	13.6−15.6	
Race/skin color^c^			< 0.001
White	24.1	23.0−25.2	
Black	25.8	23.5−28.1	
Brown	21.4	20.3−22.6	
Health insurance			< 0.001
Yes	22.8	21.9−23.7	
No	25.3	24.2−26.3	

^a^ Weighted percentage to adjust the sociodemographic distribution of the Vigitel sample to the distribution of the adult population of each city projected for 2013.

^b^ Test of independence (Chi-square).

^c^ Excluding the skin color categories yellow, red, does not know and did not inform.

Regarding lifestyle, prevalence of self-reported AH identified by Vigitel was higher among former smokers (37.4%; 95%CI 35.7–39.2), obese individuals (43.9%; 95%CI 41.8–45.9), those who do not consume fatty red meat (26.1%; 95%CI 25.3–27.0), those who do not consume alcoholic beverages (25.2%; 95%CI 24.5–26.0), and adults who self-reported lower salt intake (24.7%; 95%CI 23.9–25.5). Among the participants who were insufficiently active in the four domains of physical activity (leisure, work, transport and domestic), prevalence of self-reported AH was 29.9% (95%CI 28.5–31.2), and was lower among adults who did exercise in their free time, 18% (95%CI 17.0–19.0) (Table 2).

**Table 2:** Prevalence of self-reported high blood pressure and 95%CI in adults (aged ≥ 18 years) according to lifestyle, reported noncommunicable diseases and self-rated health status for the Brazilian state capitals and Federal District. Vigitel, 2013.

Variable	%^a^	95%CI	p^b^
Smoking			< 0.001
Nonsmoker	20.1	19.3−20.9	
Former smoker	37.4	35.7−39.2	
Smoker	21.6	19.5−23.8	
Body mass index classification			< 0.001
Normal	14.9	14.1−15.7	
Overweight	27.3	26.0−28.5	
Obesity	43.9	41.8−45.9	
Recommended consumption of fruit and vegetables (five or more days per week)			0.025
Yes	25.5	24.1−26.9	
No	23.7	22.8−24.5	
Consumption of fatty red meat			< 0.001
Yes	19.6	18.4−20.8	
No	26.1	25.3−27.0	
Alcohol abuse			< 0.001
No	25.2	24.5−26.0	
Yes	18.3	16.6−19.9	
High salt intake			< 0.001
No	24.7	23.9−25.5	
Yes	20.8	19.1−22.6	
Diabetes			< 0.001
No	20.9	20.2−21.6	
Yes	67.1	64.2−70.0	
High cholesterol			< 0.001
No	18.4	17.7−19.2	
Yes	46.3	44.5−48.0	
Self-rated health status			< 0.001
Good	16.3	15.5−17.0	
Average	36.9	35.5−38.4	
Poor/very poor	44.9	41.1−48.8	
Insufficient physical activity in the four domains^c^			< 0.001
No	21.6	20.7−22.4	
Yes	29.9	28.5−31.2	
Physical activity in free time			< 0.001
No	27.2	26.3−28.1	
Yes	18.0	17.0−19.0	

^a^ Weighted percentage to adjust the sociodemographic distribution of the Vigitel sample to the distribution of the adult population of each city projected for 2013.

^b^ Test of independence (Chi-square).

^c^ Four domains: leisure, work, transport and domestic.

Adults who reported diabetes had a prevalence rate of self-reported AH of 67.1% (95%CI 64.2–70.0), and among those with high cholesterol, prevalence was 46.3% (95%CI 44.5–48.0). Individuals who rated their health status as being poor or very poor had a prevalence rate of self-reported AH of 44.9% (95%CI 41.1–48.8) (Table 2).

In the multivariate analysis, model 1 shows the adjustment with all explanatory variables associated with the outcome. The variable “self-rated health status” showed collinearity with other variables in model 1, and was therefore removed from model 1. After adjusting for age, gender and schooling, the following variables were not associated with the outcome and were not inserted in model 2: consumption of alcoholic beverages, insufficiency in the four domains of physical activity, recommended consumption of fruit and vegetables (five or more days per week), consumption of fatty red meat, having health insurance. Skin color was classified as black and others (white and brown) (Table 3).

**Table 3:** Multiple association models between selected variables and self-reported high blood pressure in adults (aged ≥ 18 years) for the Brazilian state capitals and Federal District. Vigitel, 2013.

Variable	OR	95%CI	p
Model 1			
Race/skin color^a^			
White	1.0		
Black	1.3	1.1–1.5	< 0.01
Brown	1.1	0.9–1.2	0.10
Obesity			
No	1.0		
Yes	2.7	2.4–3.0	< 0.01
Diabetes			
No	1.0		
Yes	2.9	2.5−3.5	< 0.01
High cholesterol			
No	1.0		
Yes	1.9	1.8−2.2	< 0.01
Former smoker			
No	1.0		
Yes	1.2	1.0−1.4	0.01
High salt intake			
No	1.0		
Yes	1.2	1.0−1.4	0.02
Insufficient physical activity in the four domains			
No	1.0		
Yes	1.1	0.9−1.2	0.07
Recommended consumption of fruit and vegetables (five or more days per week)			
Yes	1.0		
No	1.0	0.9−1.1	0.87
Consumption of fatty red meat			
Yes	1.0		
No	1.1	0.9−1.2	0.26
Alcohol abuse			
No	1.0		
Yes	1.1	0.9−1.3	0.26
Health insurance			
Yes	1.0		
No	1.1	0.9−1.2	0.12
Age group (years)			
18–24	1.0		
25–34	2.6	2.0−3.4	< 0.01
35–44	5.4	4.2−6.9	< 0.01
45–54	11.1	8.6−14.3	< 0.01
55–64	18.7	14.5−24.2	< 0.01
65 or over	28.5	21.9−37.1	< 0.01
Schooling (years)			
0–8	1.0		
9–11	0.8	0.7−0.9	< 0.01
12 or over	0.7	0.6−0.8	< 0.01
Gender			
Female	1.0		
Male	0.9	0.8−0.9	0.02
Model 2^b^			
Race/skin color^a^			
White/brown	1.0		
Black	1.3	1.1−1.5	< 0.01
Obesity			
No	1.0		
Yes	2.7	2.4−3.0	< 0.01
Diabetes			
No	1.0		
Yes	2.9	2.5−3.5	< 0.01
High cholesterol			
No	1.0		
Yes	1.9	1.8−2.2	< 0.01
Former smoker			
No	1.0		
Yes	1.2	1.1−1.3	< 0.01
High salt intake			
No	1.0		
Yes	1.2	1.0−1.4	0.02
Age group (years)			
18–24	1.0		
25–34	2.6	2.0−3.4	< 0.01
35–44	5.4	4.2−6.9	< 0.01
45–54	10.9	8.6−14.1	< 0.01
55–64	18.5	14.3−23.9	< 0.01
65 or over	28.1	21.7−36.4	< 0.01
Schooling (years)			
0–8	1.0		
9–11	0.8	0.7−0.9	< 0.01
12 or over	0.6	0.6−0.7	< 0.01
Gender			
Female	1.0		
Male	0.9	0.8−1.0	0.04

Notes: Weighted percentage to adjust the sociodemographic distribution of the Vigitel sample to the distribution of the adult population of each city projected for 2013.

^a^ Excluding the skin color categories yellow, red, does not know and did not inform.

^b^ Non-significant variables in Model 1 were excluded.

Model 2 showed the final variables associated with the outcome. Adults most likely to be hypertensive were those who reported as black, obese, with a medical diagnosis of diabetes, and former smokers. There was progressive increase in the likelihood of high blood pressure with age, using the 18 to 24 age group as reference. Increase of schooling levels, on the contrary, was protective of high blood pressure. Male adults showed lower prevalence at the significance limit (OR = 0.9; 95%CI 0.8–1.0; p = 0.04), and adults who reported high salt intake had a higher probability of high blood pressure, also at the significance limit (OR = 1.2; 95%CI 1.0–1.4) (Table 3).

## DISCUSSION

The findings of this study show that about a quarter of the adult population living in the Brazilian state capitals reports having AH. The variables associated with AH were: increasing age and aging, low schooling levels, black race/skin color, obesity, self-reported diabetes or high cholesterol, being a former smoker, and high salt intake. The variable female gender was at significance limit.

Prevalence of AH observed in the 2013 Vigitel was similar to that described in Brazil and worldwide using self-reported diagnosis^7,9,d^. Among the multiple elements that contribute to determine AH are genetic factors (age, gender, family history), lifestyle (smoking, obesity, alcohol abuse, physical inactivity, stress and high salt intake), physical and psychosocial setting (stress, schooling), the organization of health services and the relationships between these various elements, some of which are shown in this study^9,10^.

In this study, women showed higher prevalence of self-reported AH, but at the significance limit after adjustment for several covariates. WHO data show that prevalence of AH among men is higher than among women, both worldwide (29.2% for men and 24.8% for women) and in the Americas (26.3% for men and 19.7% for women)^f^. Considering the findings of studies with self-reported data, women showed higher prevalence of AH^4,7,11^. The greater demand for health services by women may represent a greater opportunity for the medical diagnosis of AH^4,12^. Therefore, such higher prevalence does not necessarily indicate a higher risk of high blood pressure among women.

The relationship between AH prevalence and increasing age has been widely reported^10,a^. In the age group over 65, prevalence of AH was above 60% and may be explained by aging-specific biological alterations, such as arterial stiffening and increased peripheral vascular resistance^10,13^.

Prevalence of AH was higher among black race/skin color adults, followed by whites and browns. Among men, there was no difference according to race/skin color. Studies show higher prevalence of AH among blacks^14,15^. In Brazil, Lessa^9^ found prevalence rates up to 130% higher among black women compared to white women. In the literature, the probable factors related to higher prevalence of AH in the black population are genetic predisposition, worse living conditions, less access to health services and stress due to racial discrimination^9,g^.

No differences were found in this study related to having or not health insurance. Having health insurance may facilitate access to health services and preventive action^13^. However, the higher schooling levels among respondents with health insurance may be responsible for the loss of statistical significance in the multivariate model.

The literature evidences smoking as a strong risk factor for cardiovascular diseases^16,a^, and smoking cessation is recommended as a priority measure in the secondary prevention of cardiovascular diseases and other NCDs^16,a^. This study showed the association between smoking (former smoker) and AH. This association may be due to an effect resulting from the cross-sectional study design: former smokers may have abandoned the addiction following medical advice after the diagnosis of high blood pressure, given the harmful effects of smoking. Another explanation may be that smoking cessation triggers weight gain, increasing the risk of AH. Indeed, the association between former smokers and diabetes has already been observed in longitudinal studies, probably due to weight gain after smoking cessation caused by metabolic factors^17^, but the relationship between former smokers and AH still needs to be investigated in further studies.

Obesity is an important risk factor for high blood pressure^a,b^. This study found a positive gradient between overweight/obesity and AH prevalence. Obese people would be up to three times more likely to develop AH^18^. Thus, losing weight is the most effective method for reducing blood pressure in obese individuals, as well as contributing to the reduction of antihypertensive dosages^10^.

The consumption of five or more daily portions of fruit and vegetables is recommended by the WHO to reduce the incidence of NCDs^a,b^and prevent and treat overweight and diabetes^10,19,a^. This study did not find a direct association between the recommended consumption of fruit and vegetables and AH. A higher prevalence of AH was observed among adults who reported consuming fatty red meat. However, consumption of fatty red meat was not included in the final model. Consumption of fatty meat is recognized as a risk factor for cardiovascular diseases^a^.

Alcohol intake causes changes in blood pressure and is associated with increased cardiovascular morbidity and mortality^20^, although this association was not found in the current study.

High salt intake was associated with higher prevalence of AH at the significance limit. High salt intake is harmful to the health and is associated with AH. This relationship is partly caused by increased water retention in the body, overloading the cardiac function and potentially resulting in high blood pressure^21,a^.

Diabetes and high cholesterol also showed an association with AH among adults interviewed in the 2013 Vigitel. The association between diabetes and AH is established by pathophysiological mechanisms, and prevention and treatment of these comorbidities (AH, diabetes and high cholesterol) are essential in controlling more severe outcomes and preventing mortality^19^. Eating fatty foods increases blood cholesterol levels and also poses a higher risk of heart disease.

AH prevalence among individuals who self-rated their health as poor or very poor was almost twice as high. This may be associated with both the symptoms and the changes brought about by the disease, such as a greater number of medical appointments and visits to health services, changes in lifestyle, use of medicines and restriction of daily activities, leading to the perception of worsening health conditions^22^. However, the poor self-evaluation was not included in the final model, presenting collinearity with other variables.

In this study, participants who were insufficiently active in the four domains (leisure, work, transport and domestic) showed higher prevalence of AH, as did those who were not active in their leisure time. The importance of physical activity for the treatment and reduction of AH prevalence and, consequently, of mortality caused by cardiovascular diseases is recognized^19^; however, the variables related to physical activity were not included in the final model.

One limitation of this study is the fact that the cross-sectional design measures the event and outcomes simultaneously. As a behavior change may occur due to the event being studied, the associations herein described must be viewed with caution regarding the causal model. Another limitation is the possibility that AH prevalence is underestimated, since high blood pressure may be subclinical or undiagnosed in a large part of the population studied.

The Vigitel telephone survey has limitations, such as restricting the sample to individuals residing in Brazilian state capitals and the Federal District who have a landline phone. This is minimized by the use of data weighting factors, which seek to adjust the demographic characteristics of the Vigitel sample to the characteristics of the total population, according to population census data^e^.

Aging, black race/skin color, low schooling levels, obesity, being a former smoker, reporting diabetes and high cholesterol, in addition to high salt intake, were associated with a higher risk of AH among adults living in the 26 Brazilian capitals and the Federal District in 2013. Information from Vigitel is useful to monitor the historical series of AH prevalence and identity its associated factors, and this study may help support public policies for health promotion, surveillance and care in the Brazilian Unified Health System^23^.

## Footnotes

a World Health Organization. Global status report on noncommunicable diseases 2010. Geneva: WHO/NUT/NCD; 2011 [cited 2014 Feb 28]. Available from: http://www.who.int/nmh/publications/ncd_report2010/en/

b World Health Organization. Integrated management of cardiovascular risk: report of a WHO meeting: Geneva; 2002 [cited 2014 Feb 28]. Available from: http://www.who.int/cardiovascular_diseases/media/en/635.pdf

c Centers for Disease Control and Prevention. National Health and Nutrition Examination Survey. Atlanta; s.d. [cited 2008 Jan 20]. Available from: http://www.cdc.gov/nchs/about/major/nhanes/nh3data.htm

d Instituto Brasileiro de Geografia e Estatística. Pesquisa Nacional de Saúde 2013: percepção do estado de saúde, estilos de vida e doenças crônicas – Brasil, grandes regiões e unidades da federação. Brasília (DF): IBGE; 2014 [cited 2015 May 20]. Available from: ftp://ftp.ibge.gov.br/PNS/2013/pns2013.pdf

e Ministério da Saúde (BR), Secretaria de Vigilância em Saúde, Departamento de Vigilância de Doenças e Agravos Não transmissíveis e Promoção da Saúde. VIGITEL Brasil 2013: vigilância de fatores de risco e proteção para doenças crônicas por inquérito telefônico. Brasília, DF: Ministério da Saúde; 2014 [cited 2015 May 20]. Available from: http://portalsaude.saude.gov.br/images/pdf/2014/dezembro/09/Vigitel-2013.pdf

f World Health Organization. World health statistics 2012. Geneva: WHO; 2012[cited 2013 Feb 14]. Available from: http://www.who.int/gho/publications/world_health_statistics/2012/en/

g Instituto Brasileiro de Geografia e Estatística. Pesquisa Nacional por Amostra de Domicílios: um panorama da saúde no Brasil: acesso e utilização de serviços, condições de saúde e fatores de risco e proteção à saúde 2008. Rio de Janeiro: IBGE; 2010 [cited 2013 Feb 14]. Available from: http://www.ibge.gov.br/home/estatistica/populacao/panorama_saude_brasil_2003_2008/PNAD_2008_saude.pdf
